# Alumni Association of the American Veterinary College

**Published:** 1890-04

**Authors:** A. T. Sellers

**Affiliations:** Secretary


*Alumni Association of the American Veterinary College.*—The fourteenth annual meeting of the Alumni Association of the American Veterinary College was held in the lecture room of the college, New York City, on the morning of March 5th, 1890, the president, Dr. W. H. Hoskins, in the chair. The following members responded to roll-call: Drs. Birdsall, Chapin, Coates, Devonds, Engeman, Fagan, Flagge, Harrison, Hoskins, Huhne, Dodin, Kuehne, Lowe, Sellers, Smith, Strange, Vogt, Wright, Ridges, Miller. The minutes of the thirteenth annual meeting were read and approved.

The Executive Committee reported the purchase of the alumni prize, consisting of three books; also that they had a meeting with a committee from the graduating class in reference to their partaking of dinner with the Alumni Association, and as a result of the meeting they would banquet with the Alumni Association at Clarke's restaurant, No. 22 West 23d Street. On motion the report was received and filed.

Dr. Coates reported on behalf of the Board of Trustees that the College was in a flourishing condition, there being no better proof than the attendance of her students. This year she has the largest number of students. The graduating class is smaller this year than for the past few years, but this is owing to the fact that the majority of the students were taking a three years' course instead of two as in former years. Not many years ago the matriculation book counted more matriculants than at present, which was due to the Columbia Veterinary College closing its doors and turning over her students, books and charter to the American Veterinary College. The Board of Trustees of the College are men of intelligence and education, men of great ability employed in different pursuits of life, and fully able to meet all emergencies. By their counsel they are able to guide any corporation, and to cope with any matter brought before them. Statesmen, journalists, bankers, brokers, lawyers, doctors, are those comprising the Board. Not only that, but men who are connected with the educational institutions. So that no matter what point is brought before them there is no possible chance of the college being misguided.

Dr. Hoskins addressed the meeting and said: "I welcome all who are present to-day. I have only words of commendation for that which has been done so well, that which has been completed so nicely, that which has been attained by the alumni of the American Veterinary College. Exalted the character of work done by her noble founder, ever the same unsullied, untarnished career she has held as an educator in the ranks of veterinary science, and to-day, in all North America, she stands without a peer, and but few her equal. Chastened on every side by every example of a blighted course, competed with from very standpoint, and ofttimes by unscrupulous methods that should to-day be scorned by every fair-minded man, year by year she has surely added to her strength by new productions of such a character and value to our nation, that have increased the wealth and pleasure of our country, in the added security and safeguards we represent for her gigantic live stock interests, the truest and most substantial value of a nation. In every movement of a national, state or local character looking to the care and preservation of our live stock interests, the offspring of your alma mater have taken a deep and important part. In National Bureaus, State Boards, City Boards and Commissions, her graduates have won for themselves the highest commendations and appreciation, and in this way reflected honor and glory upon our college, that is the admiration of the world."

Dr. Sellers moved, and was seconded, that we indorse the Army Veterinary Bill. Carried.

The following was offered and ordered spread upon the minutes:

"At the annual meeting of the American Veterinary Alumni Association the aims and purposes of the House of Representatives' Bill No. 3912, relative to Army Veterinary Legislation, presented for your consideration through the committee of the Uuited States Veterinary Medical Association, was endorsed, and it was further resolved that we place on record through the President of the Senate and Speaker of the House of Representatives the action taken."

The following officers were unanimously elected to serve one year:—President, Dr. W. H. Hoskins; vice-president, Dr. Theodore Birdsall; treasurer, Dr. A. Strange; secretary, Dr. A. F. Sellers; librarian, Dr. G. A. Lathrop.

On motion we adjourned to meet again at 10.30 P. M. around the festive board, where fifty-six members of the alumni and the faculty sat down to an elegant repast, to which full justice was done by all present.

Toasts: "American Veterinary College," F. D. Weisse, M.D.; "Progress of Veterinary Medicine," A. Liautard, M.D., V.M.; "Faculty," O. D. Pomeroy, M.D.; "Sister Profession," C. A. Doremus, M.D.; "Alumni Association," W. H. Hoskins, D.V.S.; "Bureau Animal Industry," A. K. Robertson, D.V.S.; "Class of '89–'90," A. F. Becker, D.V.S.; "Class of '90–'91," E. B. Ackerman.

A. T. SELLERS, *Secretary.*

